# On the Education About/of Radical Embodied Cognition

**DOI:** 10.3389/fpsyg.2019.02378

**Published:** 2019-11-05

**Authors:** John van der Kamp, Rob Withagen, Dominic Orth

**Affiliations:** ^1^Department of Human Movement Sciences, Vrije Universiteit, Amsterdam, Netherlands; ^2^Research Center for Exercise, School and Sport, Windesheim University of Applied Sciences, Zwolle, Netherlands; ^3^Department of Human Movement Sciences, University Medical Centre Groningen, Groningen, Netherlands; ^4^Department of Health and Medical Sciences, Swinburne University of Technology, Melbourne, VIC, Australia

**Keywords:** education, motor learning, radical embodied cognition, pedagogy, ecological psychology

## Abstract

In mainstream or strong university education, the teacher selects and transmits knowledge and skills that students are to acquire and reproduce. Many researchers of radical embodied cognitive science still adhere to this way of teaching, even though this prescriptive pedagogy deeply contrasts with the theoretical underpinnings of their science. In this paper, we search for alternative ways of teaching that are more aligned with the central non-prescriptive and non-representational tenets of radical embodied cognitive science. To this end, we discuss recent views on education by Tim Ingold and Gert Biesta, which are based on Dewey’s philosophy of pragmatism and Gibsons’ ecological approach. The paper starts by introducing radical embodied cognitive science, particularly as it relates to motor skill learning, one of our prime interests in research and teaching. Next, we provide a synopsis and critique of the still dominant prescriptive and explicating pedagogy of strong education. Following Ingold and Biesta, we search for a weak alternative through a careful consideration of the education of attention and the participating teacher. To illustrate our arguments, we use examples of the first author’s teaching about/of motor skill learning. The paper is concluded by briefly considering the implications of weak education for a radical embodied science of motor skill learning.

“Seek the truth and you will not find it, knock at its door and it will not open to you, but that search will serve you in learning to do.”

Rancière (1991/2007, p. 138)

## Introduction

This paper addresses a contradictory practice among researchers of radical embodied cognition; in our teaching *about* radical embodied cognition, many of us tend to rely on the prescriptive and explicating pedagogy that squarely belongs to traditional cognitive science. On reflection, this makes us feel somewhat uneasy. Our current aim, therefore, is to search ways for the teaching *of* radical embodied cognition that are more aligned to the central non-prescriptive and non-representational tenets of radical embodied cognitive science. To this end, we discuss recent views on education as developed by Tim Ingold and Gert Biesta, among others. In particular, the thinking of Ingold, which is obviously inspired by Dewey’s philosophy of pragmatism and Gibson’s ecological approach, is of major influence. In fact, the current paper can also be read as an appraisal of Ingold’s (2018) latest, stimulating book *Anthropology and/as education*. Before addressing these perspectives on education, we first introduce radical embodied cognitive science, particularly as it relates to motor skill learning, one of our prime interests in research and teaching. Next, we provide a synopsis and critique of the still dominant prescriptive and explicating pedagogy of strong education. Following Ingold and Biesta, we then search for a weak alternative, and in so doing, we closely consider the education of attention and the participating teacher. The paper is concluded by briefly feeding some of these ideas back into a radical embodied science of motor skill learning.

## Prologue: Motor Skill Learning

Although there are several articulations of radical embodied cognitive science (Thelen and Smith, 1994; Chemero, 2009; Rowlands, 2010; Barret, 2011; Hutto and Myin, 2012; Malafouris, 2013; Gallagher, 2017), they do share the claim that human skill and learning are best understood on the scale of the relationship between a person and the environment. This strongly contrasts with traditional cognitive science that seeks to explain skill and learning by focusing almost exclusively on the person, citing internal computation and representation as causal determinants. For motor skills, these internally stored representations have been referred to as motor programs (Keele, 1968), schemas (Schmidt, 1975), and codes (Prinz, 1997), among others (see Summers and Anson, 2009). In general, a motor representation consists of a set of rules or procedures that specify—in different degrees of detail—and causally underpin the sequence of movements that form the motor skill. The representations form with practice. Fitts and Posner (1967, pp. 10–11), for example, conceived motor skill learning as the conscious assembling, structuring, and sophistication of rules (or subroutines) that get consolidated into an overall internal program. This internalization of rules releases the need for conscious control, and hence, skilled performance becomes increasingly autonomous. In other words, in traditional cognitive science, skill learning is principally about acquiring—literally, that is—internal representations (Araújo and Davids, 2011). It stands to reason that within traditional cognitive science teaching (motor), skills involve introducing the learner to the most appropriate set of rules. This has resulted in a pedagogy that is largely prescriptive (e.g., Brass et al., 2017). Practice serves to acquire the rules that are later internalized in motor representations and the teacher is the main source of information for these rules. Instruction, feedback, and modeling are used, first, to deliver the learner knowledge about the desired, ideal movement sequence of pattern, and second, to weed out any remaining aberrations from the desired movement. Accordingly, teaching is prescriptive, the “stilling in” (Ingold, 2018) of an ideal movement pattern, that is, ideal to the mind of the teacher.

Dreyfus (1992, 2008) and Dreyfus and Dreyfus (1986) criticized prescriptive skill learning theories. In particular, Dreyfus argued against the idea that instructing, feeding back, and modeling about the desired movement pattern are a precursor to the internalization of rules in a motor representation. By contrast, skill learning requires that learners, if they are to achieve higher levels of competence, must cease using the movement rules, rather than internalize them. According to Dreyfus, positing that learning involves the acquisition of internal representations is “like claiming that since we need training wheels when learning how to ride a bicycle, we must now be using invisible training wheels whenever we ride. There is no reason to think that the rules that play a role in the *acquisition* of skill play a role in its later *application*” (Dreyfus, 1992, p. xiii, italics in original). Dreyfus does not deny that instruction, feedback, and modeling play a pertinent role in skill learning. Yet, rather than being prescriptive and allowing the accumulation of knowledge about the ideal movement sequence or pattern, they serve to allow the “accumulation of experience.” That is, instruction, feedback, and modeling permit a learner to practice, such as training wheels allow a child to find out how to propel and steer the bike (Dreyfus and Dreyfus, 1986, p. 22). These ideas strongly resonate in current radical embodied cognitive science, especially within the ecological approach to (motor) skill learning (e.g., Thelen and Smith, 1994; Chemero, 2009; Newell and Ranganathan, 2010; Davids et al., 2012; Chow et al., 2016; Orth et al., 2017).

In a nutshell, within the ecological approach, skill and learning are explained without recourse to internal computation or representation (Gibson, 1966, 1979). Instead, the dynamic person-environment relationship is primary, and skill learning is understood as an increasingly improved fit between the person and the environment (Davids et al., 2012). Skill learning is not uniquely determined by personal constraints, but always in unison with environmental and task constraints (Newell, 1986). In a sense, the ecological approach holds that (new) skills *emerge* rather than that they are *acquired*. Instruction, feedback, and modeling add to the constraints under which (new) movement patterns emerge (Newell and Ranganathan, 2010; Chow et al., 2016). They have no logical priority, but function in concert with other constraints to increase adaptability of the movement patterns. Similar to other variations in constraint, instruction and the like prompt a learner to search different movement patterns to satisfy the constraints. This is associated with a (nonlinear) pedagogy that is emphatically non-prescriptive (Chow et al., 2016): rather, it points the learner to the informational richness of the situation (i.e., metastable regions), allowing new movement patterns to arise or new action opportunities (i.e., affordances) to be discovered. This is what Gibson (1966) referred to as the “education of attention.”

Proponents of the ecological approach are radical in their commitment to a pedagogy that is non-prescriptive. For example, Davids et al. (2012, p. 117) maintained that “it is futile to try and identify a common, idealized motor pattern towards which all learners should aspire (e.g., learning a ‘classical’ technique for an action).” Indeed, we are very sympathetic to that commitment in our science (e.g., van der Kamp et al., 2003; van der Kamp and Renshaw, 2015; Orth et al., 2017, 2019). Nonetheless, do we truly practice what we preach? It appears to us that, ironically, in teaching *about* radical embodied cognitive science, we often rely on the prescriptive pedagogy that we strive to avoid in our research in motor skill learning.

## The Omnipresence of Strong Education

The great American educationalist, theorist, and philosopher John Dewey^1^ characterized traditional education as a business of transmission, where knowledge and skills that have been worked out in the past are being imposed on a new generation (Dewey, 1938/2015, pp. 17–18). Accordingly, in this traditional or strong education (Biesta, 2013, 2017), the teacher is an omniscient authority who selects and communicates knowledge and skills that students are to acquire, and—supposedly—use *after* they have finished school and/or university in everyday (working) life. The teacher *prescribes* and *explicates*. To paraphrase an example of Ingold (2018, p. 4), the teacher chooses the subject matter and encodes it in some form or package that allows it to be delivered to the students with minimal distortion. The students, after unpackaging it, should then—ideally—have acquired the exact same knowledge as the teacher began with. In other words, the teacher “fills” the students with knowledge content, which they receive, rehearse, and memorize. The teacher instructs, and the students learn. Freire (2008), a radical Brazilian educational theorist and founder of critical pedagogy, refers to this prescriptive pedagogy as banking education: “Education thus becomes an act of depositing, in which the students are depositories and the teacher is the depositor” (p. 244).

Today, despite a long record of vibrant opposition (e.g., Dewey, 1938/2015; Illich, 1970/2019; Rancière, 1991/2007), this is likely still how most university teachers typically teach, knowingly and unknowingly (see, e.g., Biesta, 2006, 2017). In fact, to make the prescriptive pedagogy more effective—at least according to educational managers—teachers increasingly are to adhere to principles of constructive alignment (Biggs and Tang, 2011). Teachers (are told to) describe the intended learning outcomes, and devise teaching methods, students’ learning activities, and assessment tasks to directly address the intended learning outcomes. For example, one of us (John) coordinates and teaches a course that addresses motor skill learning. The course, called *Perceptual-motor learning*, is an elective within the 1-year master of science program *Human Movement Science: Sport, Exercise and Health* ^2^. Each year, approximately 50–60 students take the course. As the teacher, John defines (or confirms) the intended learning outcomes^3^, chooses course content, teaching and learning activities, and assessment methods. Broadly speaking, John aims to prepare students to advance theory and research on motor skill learning and apply them to societal problems. To this end, students must acquire knowledge and be able to critically evaluate current theories (including not only ecological psychology, but also more traditional cognitive approaches such as schema-theory and common coding theory), methods, and results of research. John uses a compendium of classical and current scientific papers and book chapters, the contents of which are assessed in a written exam. The course is organized in lectures and tutorials. In the lectures, John uses the compulsory literature to explicate and structure the different theories and the associated research methods—by and large, John does the talking, the students listen and make notes (hopefully). In subsequent tutorials, the students present and discuss cases from the practices of sport, rehabilitation, and physical education and thus apply theory and research results to societal problems, putting them into context. As an assessment, students produce a knowledge clip in which they elaborate one case. (The times they are changing, formerly they wrote an essay.) During the tutorials, students are meant to do the talking and thinking, but John often finds himself interrupting discussions to correct—in his view—misapprehensions of theory and methods or to further explicate. In short, despite good intentions, John’s teaching is largely prescriptive, and as such shares many features of strong education^4^. As a teacher, John makes all the choices without consulting prospective students, though he does consider the suggestions made by students in the previous year’s course evaluations. By and large, students have no say in course content, it is enforced upon them and they have to adapt to it (cf. Freire, 2008). This being said, students are invited to the lectures and tutorials; attendance is not mandatory; yet, they show up in high numbers, except when exams are approaching. Also, students do value the course and teaching highly, giving ratings of quality of course content, lectures, and tutorial of approximately 4.5 on a 5-point scale.

It is good to pause. The students’ high appreciation may merely reflect theirs and John’s adaptation to banking education. Yet, according to Freire (2008):

Implicit in the banking concept is the assumption of a dichotomy between human beings and the world: a [student] is merely *in* the world, not *with* the world or with others; the [student] is a spectator, not re-creator. In this view, the [student] is not a conscious being (*corpo consciente*); he or she is rather the possessor of *a* consciousness; an empty “mind” passively open to the reception of deposits of reality from the world outside (p. 247; italics in original).

In other words, the assumptions underlying John’s teaching—as presumably that of many colleagues—deeply conflict with the assumptions underpinning his science. Even though he emphatically tries to show students that radical embodied cognitive science deserves careful consideration, John does so by regulating the way in which they encounter it. John merely deposits it upon the students. As Ingold (2018) argues: “Insofar as such training moulds the raw material of immature humans to pre-existent design, while it might replicate the design, it serves no educational purpose whatsoever” (p. 5). This conclusion may be considered a little too sweeping, but there are good reasons to be critical toward strong education.

First, as Biesta (2013, 2017) argues, strong education overemphasizes the qualification function of education. Qualification provides students with the specific knowledge and skills needed for life after school or university, typically a particular job or profession; yet, socialization and subjectification are equally important functions of education. With socialization, students are introduced in existing ways of doing of particular groups or cultures, while with subjectification, students become autonomous and independent individuals. The one-sided bias toward qualification in strong education, presumably because it offers teachers (and managers) a high certainty about students’ learning outcomes, threatens to eradicate the socialization and subjectification functions. Instead, Biesta advocates weak education, in which the mutual interdependency of qualification, socialization, and subjectification is not only recognized but fostered, despite learning outcomes becoming less predictable. A pedagogy that solely consists of prescription and explication cannot achieve this.

A second problem with strong education was recognized by Reed (1996), who was the first to consider social institutions such as work and school from the perspective of ecological psychology. Reed was profoundly influenced by the writings of Dewey. He argued that in mainstream education, the understanding of the world that students acquire is based on secondhand experience, in which knowledge is selected, modified, packaged, and presented by teachers. That is, in schools and universities, the world is not experienced firsthand, but always through (scientific) representations of the world that are construed by others. Yet, “reading cookbooks is not the same as cooking” (Reed, 1996, p. 108). Consequently, Reed emphatically calls for an education that better balances firsthand and secondhand experience. Instead of depositing knowledge from outside, education must provide students with opportunities to actively see, feel, taste, hear, or smell the world for themselves.

We are back to square one. How can we offer such a non-prescriptive, weak education?

## The Education of Attention

Education, I argue, is not a “stilling in,” but a “leading out,” which opens paths of intellectual growth and discovery without predetermined outcomes or fixed end-points. It is about attending to things, rather than acquiring the knowledge that absolves us of the need to do so; about exposure rather than immunization. The task of the educator, then, is not to explicate knowledge […] but to provide inspiration, guidance, and criticism in the exemplary pursuit of truth (Ingold, 2018, p. ix).

This extended quote is from *Anthropology and/as education* by the social anthropologist Tim Ingold. According to Ingold, knowledge and skills cannot be isolated from life experiences (see also Dewey, 1938/2015). It is not only a thinking in the head. Instead, education is necessarily intertwined with experience—firsthand experience, that is (Reed, 1996). Ingold advocates a way of education in which students learn to attend and respond to what is going on in the world. Like the way craftspeople obtain knowledge in their engagements with materials when making artifacts. Ingold takes this literally. He narrates an occasion where he takes a class of students to the beach to weave baskets out of willow (Ingold, 2013, pp. 22–24). We are told that students first stuck willows vertically in the sand to form a frame, and then alternately weaved other pieces horizontally in and out the vertical frame, and so gradually formed an inverted cone. Ingold recounts that students were surprised by how unmanageable the willows were, sometimes even springing back and scratching arms and face. Yet, the difficulty with which they bent, also held the construction together once they were in place. The basket’s precise shape proved difficult to impose. In the end, each basket turned out in a different shape, uniquely reflecting, according to Ingold, the relations between the willow and the weaver. For example, the height of the basket was related to the weaver’s body length, because weaving the sturdy willows required engagement of the whole body. Also, the strong wind bended the vertical frame, causing the baskets to be curved to one side, depending on how close they were to the shore. Students were proud with the baskets they had made. They told that “they had learned more from one afternoon than from any number of lectures and readings: above all about what it means to make things, about how form arises through movement, and about dynamic properties of materials” (Ingold, 2013, p. 24).

Rather than transmitting or depositing knowledge in the mind of students, Ingold invites his students to truly experience firsthand, to do undergoing. Although this experience may also help students increase their knowledge *about* matter and form, and indeed is likely to do so, the main purpose is to promote students’ knowledge *of* making, basketry in this case. According to Dewey (1938/2015), the continuity of these experiences is the gist of education. Every experience, every doing undergoing requires a student to actively attend and respond to the environment, or, as Ingold (2013, 2018) phrases it, to *correspond with the world*. A continuity of experiences transforms the student, affects subsequent experiences, and opens up new environments (Dewey, 1938/2015, p. 37). In this respect, education is (also) about becoming attentive and responsive (i.e., response-able) to the world (Reed, 1996; Ingold, 2001, 2018; Masschelein, 2010). It is here that Ingold refers to the *education of attention*.

It was Gibson (1966), the originator of ecological psychology, who introduced the concept of education of attention in the context of perceptual learning. Gibson argued that, with practice, an individual comes to “orient more exactly, listen more carefully, touch more acutely, smell and taste more precisely, and look more perceptively” (p. 51). In arguing for perceptual learning to be a greater noticing of differences (cf. Dreyfus, 2008), Gibson disputed traditional theories of perception, which conceived perceptual learning as a process of enrichment. With enrichment, perceptual learning unfolds by acquiring increasingly refined representations that add meaning to sensory stimulation. Interestingly, as Gibson and Gibson (1955, p. 34) noted, enrichment theories thus hold that “perception is progressively in *decreasing correspondence with stimulation*.” In contrast, Gibson and Gibson claimed that perceptual learning is better characterized as an increasingly differentiated responsiveness to the environment, resulting from a greater correspondence with stimulation or information.

Gibson (1966, 1979) only provided us with a minute description of the education of attention^5^. Arguably, the most elaborate interpretation has been provided by Michaels and Carello (1981) and also see Ingold (2001), although it also has been expanded upon by developmental psychologists who typically refer to it as differentiation (e.g., Adolph, 2000; Gibson and Pick, 2000). Michaels and Carello capture attention as the control of the detection or pickup of information. Attention thus helps explain that different individuals or an individual at different times has a variety of perceptions; they pick up different information. It is not that representations show different activation patterns or that stimuli are processed differently, rather different information is attended to. Attention is embedded in exploratory activity, during which the individual actively searches the environment, allowing an individual to control the information that is detected. It reveals the affordances of the situation, that is, the opportunities that the environment offers the individual. Perceptual learning then becomes the education of attention: a progressive change in the control of the detection of information that correlate with the affordances of the environment. In this respect, learning does not result in an individual who has acquired new knowledge, but in an individual who inhabits a richer landscape of affordances (Rietveld and Kiverstein, 2014, see also Dreyfus, 2008). As such, “the education of attention can manifest itself in many ways. The domain covered by the phrase *education of attention* is far broader than what has been called *perceptual learning*. Indeed, we would claim that all learning can be understood as the education of attention” (Michaels and Carello, 1981, p. 81).

To educate attention thus makes experience possible; it invites students to do undergoing, perhaps revealing new affordances. Although in this opening up to the world, the searching is deliberate, what will be revealed—if anything—is not known beforehand. It is the active, inquiring, expanding experiencing that counts, not the particular content or outcome of the experience (Reed, 1996). In contrast to the prescriptive pedagogy of strong education, weak education always entails a degree of uncertainty for students in their encounters with the world. In the words of Masschelein (2010; see also Biesta, 2013), it

“invites [the student] to go outside into the world, to expose oneself, i.e., to put [her]self in an uncomfortable, weak position, and it offers the means and support to do so. I think that it offers means for experience (instead of explanations, interpretations, justifications, representations, stories, criteria, etc.), means to become attentive. These are poor means, means, which are insufficient, defective, which lack signification, do not refer to a goal or an end.”

Seemingly, this is what Ingold (2013) intended, when inviting his students to the beach for basketry, and that is what John had in mind when introducing the practical into the *Perceptual-motor learning* course, asking his students to practice a new skill. Not merely to put theory into context, but to attempt to educate the students’ attention toward …, who knows what?

In this practical, students practice one new skill of their own choice, 5 min every day over a 5-week period spanning the entire course. Students practice juggling, knitting, whistling their fingers, performing hand stand, rolling a coin across their knuckles, playing the ukulele, and many other skills. Along the way, they actively explore the affordances of skill learning: they move, imitate, try, expose themselves to errors, repeat, feel, correct, take risks, get energized, quit, plan, reflect, vary practices, think, get bored, frustrated, are proud, notice and adapt; in short, they do undergoing learning, become attentive to increasingly differentiated aspects of learning. After 5 weeks, the practical is concluded with a collective skill demonstration event. This event is merely for fun and to reflect on their practice, because it is the experience of practicing that counts, not the learning outcome. The practical is not entirely noncommittal, though. John verifies that students do undergoing by placing attentional pointers along the way (cf. Rancière, 1991/2007). These pointers are not prescriptive, but meant to initiate and continue the education of attention. They include a brief introductory lecture and weekly questionnaires, which aim to educate attention to how key theoretical concepts cognate (or not) with their practice (e.g., variability of practice, self-efficacy, modeling, degeneracy, accumulation of declarative knowledge). Actually, students suggested using vlogs instead,^6^ and hence, students now produce weekly vlogs demonstrating and reflecting on their practicing against the backdrop of a concept of their own choice. The vlogs are shared on a (protected) course website. There is a thin line between students who genuinely open up and attend and respond to how practice unfolds, and students, who, in the vlog, mostly reproduce their knowledge about the concept and use practice to exemplify. Interestingly, at the start, there is widespread enthusiasm among the students. Yet, students value the practical less than the orthodox lecturing, with ratings being consistently below 4.0. The large standard deviations perhaps indicate that some students find it difficult to handle the openness of the learning aims and/or are simply fed up with practicing after 5 weeks.

The practical is introduced by presenting the experience of Ingold’s (2013) class with basketry, emphasizing that the practical is about experiencing motor skill practice and learning firsthand. Students are made aware that attending and responding can generate knowledge, but also that this does not happen necessarily. In this respect, many students experience something akin to Bernstein’s (1967) concept of context-conditioned variability, but on a longer learning timescale. With context-conditioned variability, Bernstein voiced that the correspondence between an executive motor command and the movement depends on the context. The context is never completely fixed, but inherently variable, and further to this point, each movement changes its own context (Turvey et al., 1982; Turvey and Fonseca, 2009). Consequently, an actor cannot achieve an intended movement by generating the necessary motor command(s), because they are insensitive to the ongoing conditions. Analogously, in the practical, most students start by searching the Internet, especially YouTube, to locate tutorials that explicate and demonstrate rules about how the to-be-learned skill is to be performed, or occasionally, how it is to be practiced.^7^ Indeed, students experience following and emulating these rules as helpful, particularly to kickstart learning. However, like an executive motor command producing the movement, the tutorial instructions are largely ignorant to an individual student’s learning conditions. Accordingly, at some point, students’ attention will be drawn (or not) to the need to seek context-conditioned adaptions to further the new skill. Their attention will be educated to the need for an increasingly differentiated responsiveness, the exact nature of which they can hardly put into words, nor is it to be found in YouTube tutorials. For example, one student accumulated detailed knowledge *about* whistling with her fingers, among others, by using split screen to imitate and compare her own performance with a master model: “I am a bit stuck, and do not master whistling yet. […] I hope to see where I go wrong, because I keep level on the same thing, not being able to fully master; yet, I have no idea yet, but I hope it works.” Likely, however, her knowledge about the whistling skill was not the limiting constraint, since during the skill demonstration, her description of the rules sufficed for a fellow student to whistle almost instantaneously. Yet, the student herself found it hard to make any progress. That is, until she construed from the concept of degeneracy (Lee et al., 2014) that instead of perfecting her whistling technique, she better would start “experimenting all kinds of different manners, to find a solution that works for me, suits me best. By changing movement solutions, I was able to find a solution that worked for me, and now I am actually able to, *kind of*, whistle.”

Despite her hesitance to go beyond the certainty of explicit knowledge, the continuing failure afforded the student to start to improvise: that is, to stop using rules and accumulate differentiated experience instead (Dreyfus, 2008). Admittedly, her final performance was still best captured as hissing, but that is not the point. Over the years, the practical has afforded students this and many other firsthand experiences of motor skill learning. The content of these experiences is of less importance, what matters is that students attend to the landscape that the practical affords, that they attentively follow and respond to what happens during practice. This opening up, however, can neither be planned nor controlled:

“The aim in [practice]^8^ is not to test a preconceived hypothesis but to open a path and follow wherever it may take you. It is not so much iterative as itinerant: a journey undertaken rather than a cycle of returns on a fixed point. It works more by intuition than by reason; opening from within rather than penetrating from without. It is prospective rather than confirmatory. The patience of [practice], in this sense, lies in the dynamics of attention, and in the endurance of waiting. We have to allow things to come into presence, in their own time: they cannot be forced.” (Ingold, 2018, p. 41)

## The Participating Teacher

The teacher evidently has a role to play in educating students’ attention. Weak education does not imply the end of teaching or that an ignorant teacher would suffice (cf. Rancière, 1991/2007). Then what is the teacher’s role? One influential suggestion is that the teacher should act as a facilitator, scaffolding the learning process and creating environments that provide learning opportunities. This is the perspective of constructivism (Palincsar, 1998; Richardson, 2003). Basically, constructivism is a theory of learning, not teaching. Although many perspectives proffer, two main accounts can be distinguished (Palincsar, 1998). The cognitive or Piagetian account holds that students actively construct new understandings, learn by actively merging previously acquired knowledge with new information. Learning, then, is largely an individual process, occurring within the student and resulting in cognitive structures. In the sociocultural or Vygotskyan account, knowledge is co-constructed in collaborative activities with others, emphasizing that learning is primarily a social activity. Consequently, in constructivism, a teacher’s role is not to transmit knowledge, but rather to offer opportunities for students to create their own new knowledge, preferably in collaborative activities (i.e., iterative cycles of interaction, negotiation, and collaboration). For example, a teacher can facilitate a debate of ideas, in which students discuss a topic with the purpose to achieve a shared understanding. The teacher can enrich the debate with video-material, but does not herself participate in the discussion (Koekoek et al., 2019).

Some constructivists, particularly among those who adhere to the cognitive account, tend toward invoking a minimum role for the teacher, that is, only as a promotor of co-construction rather than as a participant (cf. Stetsenko, 2017). Biesta (2013), following Dewey, refers to this practice as *learning*, which he distinguishes from *education*. In education, both students and teacher must participate, and attend and respond to each other. Education only occurs if the activities of students and teacher mutually influence each other, open up to each other. Both sides must share a stake in the outcome and be willing to transform their ideas, understanding, and emotions. Accordingly, teachers must also be willing to expand their experiences. This can be uneasy and disturbing, but it is essential of weak education that the teacher is also present and is prepared to put what he or she has, indeed what he or she is, “on the table” (Ingold, 2018, p. 52). In fact, Biesta (2013) asserts that the teacher, in doing so, can add something that goes critically beyond what students can achieve on their own. This is the gift of teaching, but only when the students receive it as such. The gift of teaching is not something that *is*, but something that *makes a difference* ^9^ (Biesta, 2013, p. 85). It is a shared experience, perhaps in the form of making a critical or supportive remark, or posing a question, exchanging ideas, by offering guidance or inspiration, or by setting an example. It is distinctive of weak education, however, that the teacher cannot plan this gift, because planning (prescribing) it would annihilate the uncertainty that is necessary for it to be received as a gift. This is the beautiful risk of education (Biesta, 2013). All the teacher can do is expose him- or herself, and wait, the gift cannot be forced upon the student.

These are big words. The ongoings in the practical are more modest. John does himself participate. Over the years, he did practice, both successfully and in vain, silly walks, the *kendama*, a three-ball cascade in juggling, the handstand, and unicycling. John shares his practice experiences with his students, also by recording his own, fairly clumsy vlogs. This is not a process of transmission, but one of attending and responding. Also, students have stories to tell. For John, and presumably for students alike, this mutual attending and responding occasionally has pointed to alternative ways of practicing offering new perspectives of learning. Probably the most conspicuous is the emotion of learning, also because it has been underplayed in contemporary theories of motor skill learning, including theories of radical embodied cognition (for exceptions, see Colombetti, 2014; Headrick et al., 2015; Withagen, 2018). Yet, motor skill learning is circularly constrained by or nested within emotions and moods (Balagué et al., 2019). Nonetheless, researchers in motor skill learning often oversee or suppress emotion from their studies; if anything, the typical participant in an experiment is feeling uninterested. When trying to imitate John Cleese’s silly walks, John started practicing at home in the living room. Yet, after the first few successful strides, John quickly discovered that to further progress, more space was needed, which was only to be found outside, in the cold evenings, after dark. It was the only way to avoid the embarrassment of walking into one of the neighbors—almost. Commitment to practice quickly dropped and excuses to skip the day’s practice were easily found. Also, students did often suggest that feelings of commitment can positively energize and facilitate investment in practice, stimulate improvisation and/or variability during practice, and make it easy to ask important others for feedback. Nonetheless, feelings of commitment can (suddenly) give away to feelings of monotony or even avoidance, resulting in solitary, routine-like repetitive practice. Indeed, the emotion of learning may be intimately connected with accelerations and arrests in learning, respectively. But not entirely. Arrests in learning can also go together with feelings of anxiousness. John, for example, got stuck after successfully throwing and catching one and two balls while attempting to master juggling. Intriguingly, while this may seem easy enough, it felt frighteningly difficult to throw the third ball. Possibly, John was overthinking and monitoring too many rules and worried too much to risk the uncontrollable (i.e., throwing the third ball would surely mean dropping them all). In the end, merely concentrating on the rhythm of the throws, however, did do the job. It turned attention to Dreyfus’ proposition that if one keeps on seeking the safety of rules, learning will not progress (Dreyfus and Dreyfus, 1986; Dreyfus, 1992). Taking risks of failure and ceasing to use rules may go together with strong feelings of anxiety, just like succeeding can be deeply rewarding, as John can attest. Dreyfus is now mandatory reading in the course (no pun intended).

## Epilogue: Motor Skill Education

We have advocated weak education as way of teaching that is more in line with radical embodied cognitive science, and thus ecological psychology, than the widely accepted prescriptive and explicating pedagogy that characterizes strong education. In particular, we have pointed toward the necessity of educating students’ attention and the actively participating teacher to bring about a weak pedagogy. To wrap up this paper, we briefly consider the significance of weak education for a radical embodied cognitive science of motor skill learning.

A pertinent implication of weak education is that research into motor skill learning should much more emphatically address values wider than the current narrow focus on the “technics” of motor skill learning, such as the ongoing emphasis on optimizing training interventions and performance monitoring changes (Farrow and Robertson, 2017). We are not arguing that this is undesirable, but in sports, physical education, and rehabilitation, however, motor learning typically has fulfillments that go beyond qualification *per se*. For example, athletes invest many hours of practice to achieve distant goals, and in so doing, learn to persevere also when there is no immediate gratification (i.e., subjectification). Or, students discover ways of doing in sports or dance cultures they would not have encountered without physical education (i.e., socialization). In this respect, it seems appropriate to refer to motor skill *education*, rather than learning.

Motor skill education would, among others, promote expanding firsthand experience, emphasizing the accumulation of experience instead of knowledge (Dreyfus, 2008). It would challenge individuals to actively explore the “unknown” by exposing them to situations where new affordances can be created and discovered (see Dicks et al., 2017; Walinga et al., 2018). Individuals are not trained to adopt ideal movement patterns (or, for that matter, ideal gaze patterns), but they are brought into (variable) practice conditions in which numerous adaptive actions can emerge—if they feel sufficiently safe to explore. Secondly, and crucially, motor skill *education* would also strongly encourage a participating or co-adapting teacher (or coach or physiotherapist) to create opportunities for shared experiences with the learner. For example, we have recently argued that motor skill learning in sports is best captured as a process that is distributed across the athlete and the coach (Orth et al., 2019), in which athlete and coach mutually constrain each other. For instance, the coach must closely monitor variations in an athlete’s actions and emotions during practice, which can signal emerging changes in skill (Headrick et al., 2015). A coach shapes the practice conditions to enable this change. Yet, the change can never be fully orchestrated by the coach, not in the least, because the coach’s actions and emotions themselves co-constitute the practice conditions. This limited certainty and controllability in the mutual attending and responding between athlete and coach in particular affords new and creative actions. It is exactly for this reason that we should cherish weak education, both in motor skill education itself and in the education of motor skill education.

## Author Contributions

JK, RW, and DO contributed to the conceptualization and background research. JK drafted the first version of the paper and finalized the paper. RW and DO made critical revisions to the first draft.

### Conflict of Interest

The authors declare that the research was conducted in the absence of any commercial or financial relationships that could be construed as a potential conflict of interest.

## Footnotes

^1^Interestingly, Dewey was also interested in motor skills. In a paper on the reflex-arc, he argued strongly against, very much in spirit with current radical embodied cognition, the hypothesis of an independent “agency in the center of the soul” (or central idea) or a “superior force in the stimulus” as the cause of a motor response (Dewey, 1896, p. 364). Both Chemero (2009) and Gallagher (2017) identify Dewey as one of the forbearers of radical embodied cognitive science, while Lobo et al. (2018) discuss Dewey’s direct influence on ecological psychology.

^2^https://www.vu.nl/nl/studiegids/2018-2019/master/g-j/human-movement-sciences/index.aspx?view=module&origin=51003848&id=50044209

^3^In fact, John has no complete freedom in defining the intended learning outcomes. They must fit within the program objectives and exit qualifications.

^4^To John’s immediate defense, an increasingly important part in the course is the practical, during which students practice a new motor skill. It is deliberately less prescriptive and explicative. We turn to that below.

^5^In his 1979 book, education of attention did not even make the subject index. Perhaps, Gibson only meant his readers to become attentive of it.

^6^John had asked a group of students, who had finished the *Perceptual-motor learning* course, to make a proposal for revising the course. The students did this as part of an assignment to acquire a qualification for teaching in higher education. This turned out be very fruitful and inspiring. Another way to give the students a bigger say in the course contents would be to solicit their aspirations before the start of the course.

^7^In this respect, the introduction of the Internet has greatly affected how people learn new motor skills. For researchers, there is a wealth of issues to be addressed on how Internet shapes (self-controlled) motor learning. For example, as one reviewer noticed, the YouTube tutorials need not necessarily function to prescribe movement rules, but may also function as an initial reduction in the degrees of freedom, helping the students to begin practice (see also Dreyfus, 1992).

^8^In this quote, Ingold describes patient experimentation, instead of practice. Patient experimentation is contrasted with the scientific method in major science, in which knowledge is acquired from carefully planned and controlled experiments in which theory-derived hypotheses are tested. Typically, the observations and measurements in the experiment result in the refinement (and sometimes rejection) of the hypotheses, which are further tested in a subsequent experiment and so on.

^9^This is too nice not to mention. In German “to make a difference” is “schillen” and in Dutch it is “verschillen.” Ingold (2018, p. 42) explains that the English “skill” originated from “schillen” (Ingold, 2018, p. 42). Accordingly, the gift of teaching is something that creates skill.
